# Influence of misfit dislocations on the phase transition of iron(iii) oxides

**DOI:** 10.1039/d4na01061b

**Published:** 2025-06-09

**Authors:** Van-Hien Hoang, Nam-Suk Lee, Heon-Jung Kim

**Affiliations:** a Department of Physics, Graduate School, Daegu University Gyeongbuk 38453 Republic of Korea; b National Institute for Nanomaterials Technology (NINT), Pohang University of Science and Technology (POSTECH) Pohang 37673 Republic of Korea nslee@postech.ac.kr; c Department of Materials-Energy Science and Engineering, College of Engineering, Daegu University Gyeongbuk 38453 Republic of Korea hjkim76@daegu.ac.kr

## Abstract

Dislocations commonly occur in thin films under large misfit strain due to the accumulation of strain energy, significantly altering the films' properties. This study investigates the microstructure of Fe_2_O_3_ polymorphs in films of various thicknesses deposited on yttria-stabilized zirconia (001) substrates. The results reveal that the ε-Fe_2_O_3_ phase is formed and stabilized at thicknesses below a critical threshold of 20 nm. Beyond this threshold, the volume fraction of the ε-Fe_2_O_3_ phase decreases, and the α-Fe_2_O_3_ phase begins to emerge. With further increases in thickness, the ε-Fe_2_O_3_ phase fully transforms into the α-Fe_2_O_3_ phase. Detailed analysis suggests that this phase transformation is driven by the formation of misfit dislocations at the film/substrate interface, which compensates for the tensile strain induced by the substrate.

## Abstract 2

Dislocations commonly occur in thin films under large misfit strain due to the accumulation of strain energy, significantly altering the films' properties.

## Graphical abstract

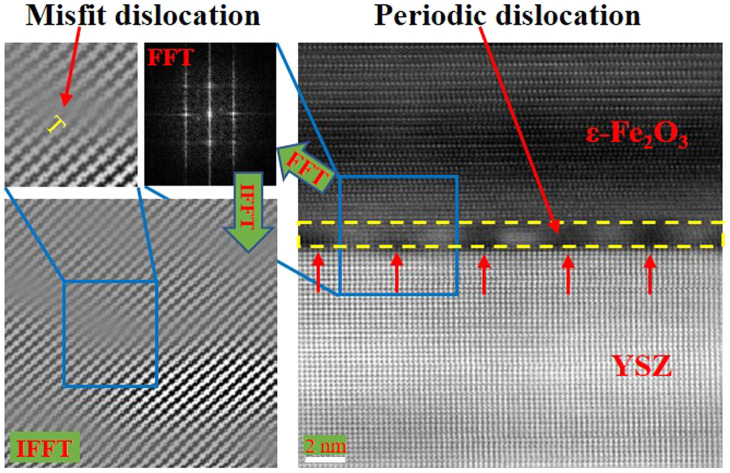

## Introduction

Dislocations are an inevitable type of defect commonly found in misfit conventional solid thin films, playing a crucial role in strain relaxation and significantly influencing the physical properties of these materials.^1–4^ These defects can act as channels for rapid ion transport,^5,6^ induce flexoelectric polarization in SrTiO_3_ (STO), and even alter the magnetic properties of materials, generating ferromagnetism in antiferromagnetic NiO^7^ and paramagnetism in ferromagnetic La_0.7_Ca_0.3_MnO_3_.^8–10^ In ferroelectric materials, dislocations cause the formation of depolarizing fields around the dislocation core, which can degrade material performance.^11^ The far-reaching impact of dislocations highlights their critical importance in both the design and functionality of advanced materials. Dislocations typically form when the film thickness exceeds a critical threshold.^1,12,13^ In such cases, film thickness acts as a macroscopic parameter that regulates stress distribution in solid films, thereby influencing their stress-sensitive structural and behavioral characteristics. This suggests that the properties of thin films are strongly influenced by their thickness, a hypothesis supported by experimental data,^14–16^ which shows that both the crystalline structure and the physical properties of films are highly sensitive to variations in thickness.

To date, four crystalline polymorphs of Fe_2_O_3_ (also known as iron(iii) oxide or ferric oxide) have been explored, each with significantly different structural and magnetic properties: α-Fe_2_O_3_, β-Fe_2_O_3_, γ-Fe_2_O_3_, and ε-Fe_2_O_3_.^17^ Among these polymorphs, ε-Fe_2_O_3_ is particularly remarkable because it exhibits a giant coercive field of approximately 2 T at room temperature. Recent studies suggest that this high room-temperature coercivity arises from the disordered structure of ε-Fe_2_O_3_.^18^ Additionally, ε-Fe_2_O_3_ exhibits a large magnetocrystalline anisotropy, which is attributed to the establishment of a single-domain character in ε-Fe_2_O_3_ nano-objects, as well as the nonzero orbital component of the Fe^3+^ magnetic moment, contributing to strong spin–orbit coupling.^19,20^ These unique properties make ε-Fe_2_O_3_ an attractive material for high-coercivity recording media. Moreover, its millimeter-wave ferromagnetic resonance and magnetoelectric coupling^21,22^ make it suitable for various applications, including electric/magnetic field-tunable devices and technologies requiring effective suppression of electromagnetic interference and stabilization of electromagnetic transmission. The ε-Fe_2_O_3_ phase could become one of the most important functional magnetic materials if synthesized in pure form and with high yield. Its practical application could potentially surpass the current material limits in technologies requiring significant magnetic hardness. Moreover, ε-Fe_2_O_3_ may open up new technological areas, benefiting from its remarkable coupled magnetoelectric properties, where an applied electric field can influence its magnetic characteristics. This property is highly valuable for applications in low-power spintronic devices, such as magnetoelectric random-access memory (MeRAM) and voltage-controlled magnetic tunnel junctions, as well as for ferromagnetic resonance capabilities, which are useful for high-frequency applications such as microwave filters, isolators, and circulators.^23^ These properties make it an ideal candidate for high-speed spintronic devices and radar communication systems, which are uncommon in simple iron oxides. However, synthesizing pure samples of this nanomaterial without contamination from other iron oxide phases is highly challenging due to its high surface energy. The high surface energy of ε-Fe_2_O_3_ results in a higher nucleation barrier, making it difficult to initiate and sustain its growth as the dominant phase. Instead, competing phases like α-Fe_2_O_3_ and γ-Fe_2_O_3_, which have lower nucleation barriers, tend to form more readily. Additionally, ε-Fe_2_O_3_ has significant thermal instability.^19,24^ Up to now, ε-Fe_2_O_3_ has been synthesized nanoparticles having a spherical (sphere-like)^25–28^ and nanorod (nanowire)morphology.^20,21,29,30^ For films, ε-Fe_2_O_3_ was successfully deposited on STO (111),^31^ yttrium-stabilized zirconia (YSZ) (100),^32,33^ and muscovite (mica)^34^ substrates. However, the mechanism underlying the stability of the ε-Fe_2_O_3_ film on the substrate remains under debate, and its phase transformation kinetics are still not fully understood. In this study, we have investigated the effect of film thickness on the film's structure. Based on X-ray diffraction (XRD) and high-resolution transmission electron microscopy (HRTEM) measurements, we demonstrate that the Fe_2_O_3_ phase transformation is attributed to the formation of a misfit dislocation array at the film/substrate interface when the film thickness exceeds a threshold of 20 nm. Note that our study focuses on specific PLD growth conditions, annealing, and the use of a YSZ substrate. Since these factors were crucial for obtaining single-phase ε-Fe_2_O_3_, variations in temperature, *p*O_2_, substrate type, and annealing conditions could influence phase formation. These insights extend beyond our specific experimental setup. However, previous studies on ε-Fe_2_O_3_ synthesis under diverse conditions also emphasize phase changes with thickness. These findings highlight that controlling misfit dislocations and phase transformations through thickness adjustments offers a pathway to tune the material properties of Fe_2_O_3_ films finely.

## Experimental section

Iron(iii) oxide thin films with varying thicknesses were deposited on a YSZ (001) substrate using the pulsed laser deposition (PLD) technique with a commercial polycrystalline α-Fe_2_O_3_ target powder. The target was positioned 5.3 cm from the substrate. A Kr excimer laser with a wavelength of 248 nm was employed, operating at a repetition rate of 2 Hz and focused to an energy density of 1.8 J cm^−2^. The deposition was conducted at 800 °C and an oxygen partial pressure of 3 × 10^−3^ torr. Following deposition, the samples were annealed by cooling to a substrate temperature of 600 °C under an oxygen pressure of 30 torr over the course of one hour. Subsequently, they were cooled to room temperature at a rate of 5 °C per minute, maintaining the same oxygen pressure.

The crystal structure of the films was analyzed using X-ray diffraction (XRD) with synchrotron radiation. Measurements were conducted at the 3A beamline of the Pohang Light Source (PLS) in South Korea, utilizing an 11.17 keV photon beam with a wavelength of 1.11 Å.

To investigate the thickness, microstructure, and atomic structure of the films, high-resolution transmission electron microscopy (HRTEM) was performed using a Cs-corrected scanning transmission electron microscope (STEM, JEOL JEM-2100F) at an accelerating voltage of 200 kV. The chemical composition of the films was assessed through energy-dispersive X-ray spectroscopy (EDS) elemental mapping.

## Results and discussion

**Fig. 1:**
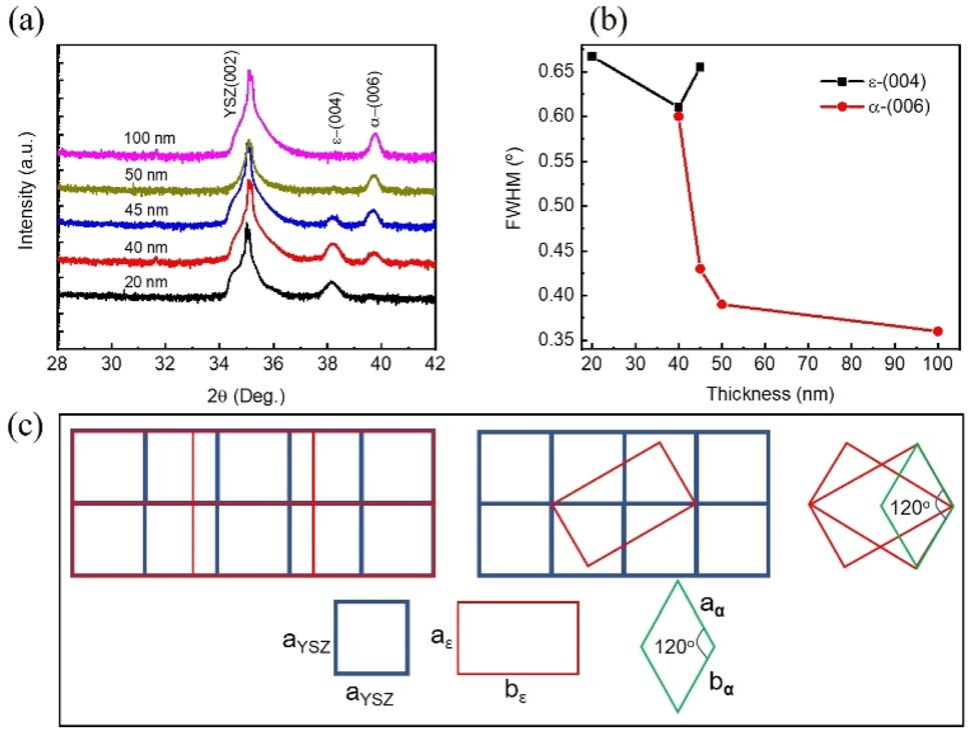
(a) XRD scans near the (002) reflection for samples of varying thicknesses. Curves are shifted vertically for clarity. (b) Full width at half maximum (FWHM) of the ε-(004) and α-(006) peaks as a function of thickness. (c) Schematic representation of the growth of ε-Fe_2_O_3_ on the YSZ (001) substrate and α-Fe_2_O_3_ on ε-Fe_2_O_3_.

Fig. 1(a) shows the *θ* − 2*θ* scans near the (002) reflection of five film samples of varying thicknesses grown on YSZ (001). The thickness of each sample, ranging from 20 nm to 100 nm, is noted on the corresponding curves. The (004) peak for the ε-Fe_2_O_3_ and (006) peak for α-Fe_2_O_3_ at 2-theta values of 18.1° and 39.7°, respectively, are clearly observed without any impurity present. According to XRD data, the ε-Fe_2_O_3_ phase is stabilized on the YSZ (001) substrate up to 20 nm. In the 40 to 45 nm range, both ε-Fe_2_O_3_ and α-Fe_2_O_3_ phases coexist. When the film thickness increases to 50 nm, only the α-Fe_2_O_3_ phase is observed. Fig. 1(b) presents the full-width at half-maximum (FWHM) of the (004) and (006) peaks as a function of thickness. While the width of (004) peaks in a small variation with the thickness, the FWHM of (006) peaks decreases monotonically as the thickness increases.

YSZ is cubic in its bulk form with a lattice parameter of *a* = 5.12 Å, while ε-Fe_2_O_3_ has an orthorhombic structure with *a*_ε_ = 5.08 Å; *b*_ε_ = 8.78 Å; *c*_ε_ = 9.47 Å. In contrast, α-Fe_2_O_3_ possesses a rhombohedral structure with *a*_α_ = *b*_α_ = 5.05 Å; *c*_α_ = 13.74 Å. Understanding how ε-Fe_2_O_3_ and α-Fe_2_O_3_ can grow on the YSZ (001) substrate is intriguing. Luca Corbellini *et al.* proposed two possible mechanisms for the ε-Fe_2_O_3_ growth on YSZ.^32^ The first mechanism involves the matching of the in-plane lattice parameter *a*_ε_ of ε-Fe_2_O_3_ with that of the YSZ substrate, as illustrated in the lower left panel of Fig. 1(c). Since *a*_ε_ is smaller than *a*_YSZ_, the film is tensile strained by the substrate to be 0.58%. In the second case, shown in the middle of the lower panel of Fig. 1(c), the diagonal of the orthorhombic unit cell, formed by the short and long sides (*a*_ε_ and *b*_ε_), matches twice the lattice constant of the substrate, 2*a*_YSZ_ ≈ (*a*_ε_^2^ + *b*_ε_^2^)^0.5^ resulting in a tensile strain of approximately 0.97%. When the film thickness exceeds 20 nm, the α-Fe_2_O_3_ phase begins to appear alongside ε-Fe_2_O_3_. There is an edge point where two ε-Fe_2_O_3_ planes form an angle of ∼120°. There is a key point where two ε-Fe_2_O_3_ planes form an angle of ∼120°, which corresponds to the angle formed in the α-Fe_2_O_3_ structure.^32^ The lower right panel of Fig. 1(c) illustrates the mechanism responsible for the formation of the α-Fe_2_O_3_ phase.

**Fig. 2:**
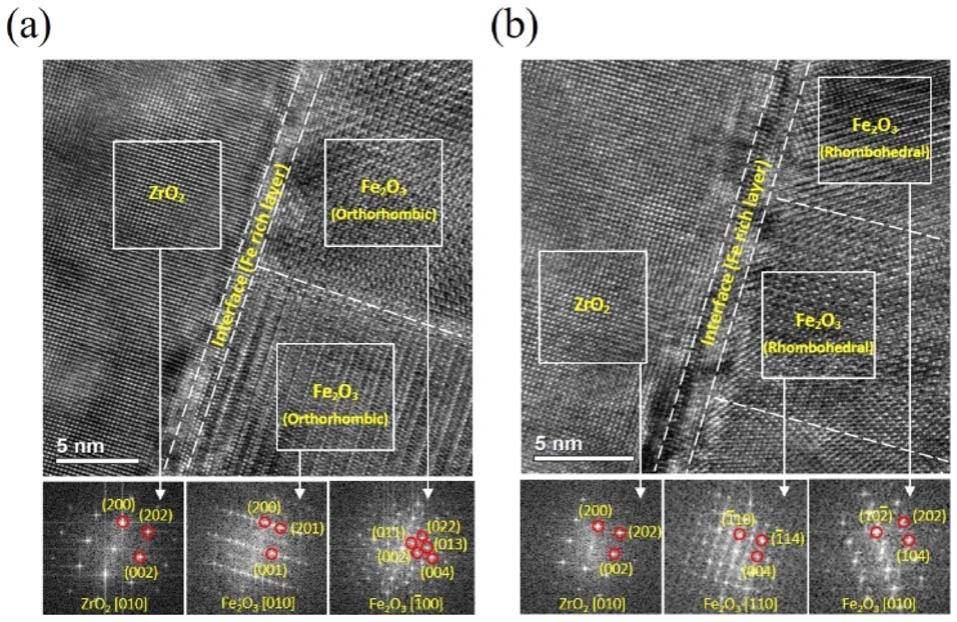
HR-TEM image of the near film/substrate interface of the samples with thicknesses of (a) 20 nm and (b) 50 nm, projected along to [100] zone-axis. Fast Fourier transform (FFT) patterns of the areas marked with the white solid boxes are on the bottom.

Fast Fourier transform (FFT) analysis of the HRTEM images was performed to examine the crystal structure and lattice orientation of the films. Fig. 2(a) and (b) show cross-sectional HRTEM images near the interface for samples with thicknesses of 20 nm and 50 nm. The corresponding FFT images of the regions highlighted by white solid boxes are displayed at the bottom of Fig. 2. The FFT analysis reveals that the crystal structure of the thickness 20 nm corresponds to an orthorhombic phase at both [010] and [−100] zone axes, consistent with ε-Fe_2_O_3_, while the film with 50 nm exhibits a rhombohedral structure at the [110] and [010] zone axes, characterized by α-Fe_2_O_3_ phase. These results are consistent with the X-ray diffraction patterns shown in Fig. 1(a). Additionally, we have determined the lattice spacing of several planes of ε-Fe_2_O_3_ near the film/substrate interface as follows: (01−3): 0.265 nm (ref. ∼0.296 nm); (−21−3): 0.177 nm (ref. ∼0.193 nm); (−200): 0.251 nm (ref. ∼0.254 nm).

Next, we will discuss why ε-Fe_2_O_3_ can be stabilized on YSZ (001) substrate up to 20 nm in thickness. For nanoparticles, in general, two factors have been found to play an essential role in determining which nanosized Fe_2_O_3_ polymorph will be formed from a given precursor and how it can subsequently be transformed into various ferric oxide phases.^35^ These parameters include chemical potential (*η*) and surface energy (*σ*). In this case, ε-Fe_2_O_3_ can exist when the size of the Fe_2_O_3_ particle falls within an interval defined by −6*ν*(*σ*_ε_ − *σ*_γ_)/(*η*_ε_ − *η*_γ_) < *d* < −6*ν*((*σ*_ε_ − *σ*_α_)/(*η*_ε_ − *η*_α_)). Where *ν* is molar volume; *d* is the size of a nanoparticle; *η*_α_, *η*_γ_, *η*_ε_ and *σ*_α_, *σ*_γ_, *σ*_ε_ are chemical potential and surface energy of α-Fe_2_O_3_, γ-Fe_2_O_3_, and ε-Fe_2_O_3_ phases, respectively. This implies that if nanoparticles of Fe_2_O_3_ grow large enough, the existence of ε-Fe_2_O_3_ is no longer favored. In other words, reducing the sizes of the Fe_2_O_3_ particle increases the contribution of the surface (or interface) energy to the system, which stabilizes ε-Fe_2_O_3_ in the nanoscaled size. However, for thin films, in addition to the two factors mentioned, strain energy contribution can play an important role in the stability of ε-Fe_2_O_3_ film. Since the YSZ substrate induces tensile strain in the films, as shown in Fig. 1(c), the relaxation of strain in the film occurs through the formation of dislocation, leading to misfit dislocation at the film/substrate interface, and the dislocation density of partially relaxed films may depend on the layer thickness.

**Fig. 3:**
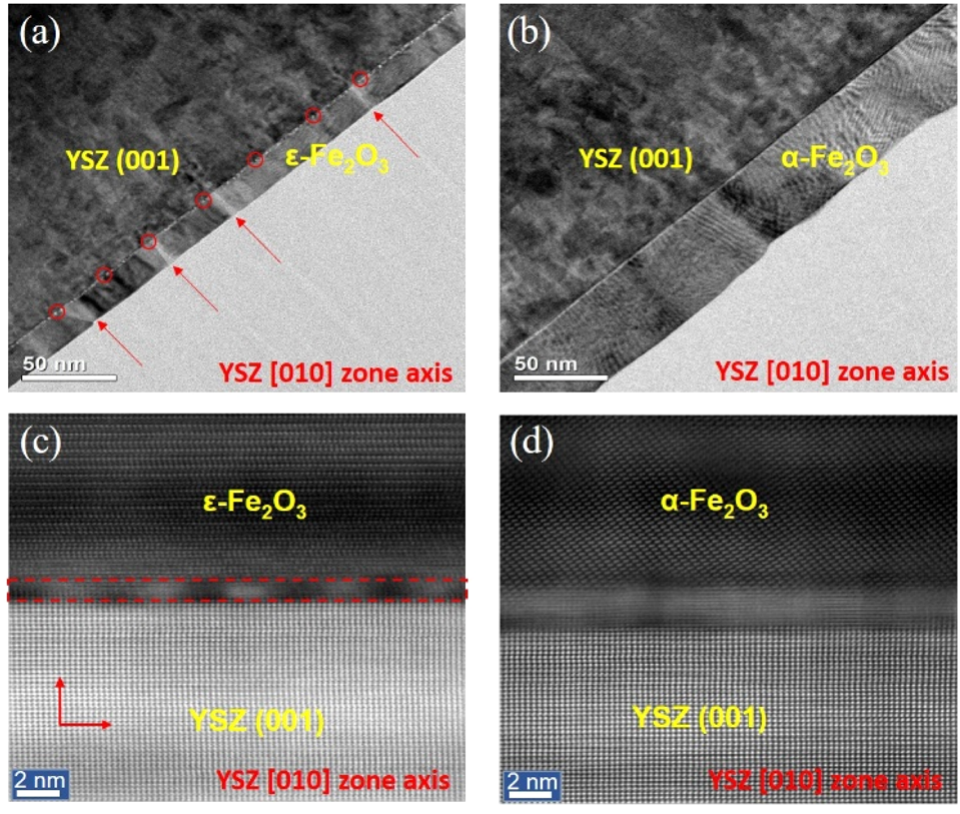
TEM image of samples across film/substrate interface with thicknesses of (a) 20 nm and (b) 50 nm, projected along the [100] zone axis. The red arrows and circles indicate threading and misfit dislocations, respectively. (c and d) Cross-sectional HAADF-STEM images corresponding to (a and b). The red dashed rectangle in (c) highlights the periodic misfit dislocations.

To confirm the existence of dislocations, the microstructure of the as-prepared thin films with thicknesses of 20 nm and 50 nm on the YSZ (001) substrate was investigated using a high-angle annular dark-field scanning transmission electron microscopy (HAADF-STEM) projected along the [100] zone axis, as shown in Fig. 3. Fig. 3(a) reveals periodic black dots at the ε-Fe_2_O_3_/YSZ interface (marked by red circles) and straight lines (indicated by red arrows) corresponding to misfit dislocations and threading dislocations, respectively.^5,31,36^ Since there is a large lattice mismatch between the ε-Fe_2_O_3_ thin films and the substrate, initially, the film remains coherently strained, meaning its lattice conforms to the substrate. However, as the film grows thicker, the accumulated strain energy increases. Once the critical thickness is exceeded, the film can no longer maintain coherent strain, and misfit dislocations form at the interface to relieve strain. This transition leads to a strain-relaxed state, where the film begins to adopt its bulk crystal structure rather than being constrained by the substrate lattice. As a result, the film loses its coherency with the substrate.^11^ In our study, the observed structural transition at increased film thickness is a consequence of this strain relaxation mechanism. As the film thickness increases to 40–45 nm (with dislocations still observed in the HAADF-STEM images, though not shown here), the density of misfit dislocations rises, generating stress fields that effectively compensate for the misfit stresses. If it is energetically favorable for misfit dislocations to form at the film/substrate interface, these defects on the ε-Fe_2_O_3_ surface may act as catalysts for the nucleation of a new phase, α-Fe_2_O_3_, within the film.^37^ This is consistent with the observed data in Fig. 1(a), where ε-Fe_2_O_3_ and α-Fe_2_O_3_ phases coexist when the thickness is within the range of 40–45 nm. The role of misfit dislocations as catalytic nucleation sites for the formation of a new phase has also been observed in other materials.^4,14,38^ The relative potency of these catalytic sites determines the spatial distribution of nucleation sites. Once the misfit dislocation network is fully formed as dislocation arrays at the film/substrate interface, the process of climb occurs, characterized by mass transportation around the dislocation cores. This relaxes the misfit strain in the film layer near the free surface, bringing the crystal lattice parameter of this layer closer to that of its ideal, unstrained state. Consequently, when the thickness reaches 50 nm, the strain impact from the substrate on the ε-Fe_2_O_3_ film becomes negligible, resulting in the instability of ε-Fe_2_O_3_ and its complete transformation into the α-Fe_2_O_3_ phase. Interestingly, in this case, dislocations completely disappear in the α-Fe_2_O_3_ film, as shown in Fig. 3(b). The behavior of the misfit dislocation becomes even clearer when viewed on a larger scale in the HAADF-STEM images shown in Fig. 3(c) and (d), where periodic black dots are observed at the ε-Fe_2_O_3_/YSZ interface [Fig. 3(c)], but are absent at the α-Fe_2_O_3_/YSZ interface [Fig. 3(d)].^36^ The absence of misfit dislocations in the 50 nm-thick film (α-Fe_2_O_3_) can be explained by calculating the misfit strain energy density (*w*^f^) accumulated in the film, which is induced by the substrate. According to M. Y. Gutkin *et al.*, *w*^f^ can be described as follows:^4^

*w* ^f^ = [*G*/(1 − *v*)](*f*_a_^2^ + *f*_b_^2^ + 2*vf*_a_*f*_b_), which can be simply rewritten as

1 *w*^f^/*G* = (*f*_a_^2^ + *f*_b_^2^ + 2*vf*_a_*f*_b_)/(1 − *v*),

where *G*, *v* are the shear modulus and Poisson ratio of the film; *f*_a_ = (*a*_f_ − *a*_s_)/*a*_f_ and *f*_b_ = (*b*_f_ − *b*_s_)/*b*_f_ represent the misfit strains induced by the substrate along the in-plane lattice parameters *a*_f_ and *b*_f_, respectively; *a*_s_ and *b*_s_ are the in-plane lattice constants of the substrate. To evaluate the strain relaxation behavior in ε-Fe_2_O_3_ and α-Fe_2_O_3_ films, we estimated the in-plane misfit strain energy density using a normalized form *w*^f^/*G*, as given in eqn (1). While the strain energy density *w*^f^ generally depends on both the shear modulus *G* and Poisson's ratio *v*, we assume that these elastic constants are approximately similar for both phases. This assumption allows for a qualitative comparison of misfit strain energy densities between ε-Fe_2_O_3_ and α-Fe_2_O_3_. Using *v* = 0.31 for both, we estimate the elastic energy densities for each phase based on the formula (1) as follows: *w*^f^_ε_/*G* = 0.245 for the ε-Fe_2_O_3_ phase, and *w*^f^_ε_/*G* = 1.189 × 10^−3^ for the α-Fe_2_O_3_ phase. The misfit strain energy density of α-Fe_2_O_3_ is much smaller compared to that of ε-Fe_2_O_3_. Since the accumulation of misfit strain energy density in the 50 nm-thick α-Fe_2_O_3_ film induced by the substrate is minimal, the formation of misfit dislocations is not necessary to compensate in this case. Additionally, this relative difference helps explain why ε-Fe_2_O_3_ exhibits a higher density of misfit dislocations and a thinner interfacial layer, whereas α-Fe_2_O_3_ relaxes more gradually through a broader interface [see Fig. 3(c) and (d)]. Furthermore, due to the high lattice misfit between ε-Fe_2_O_3_ and the substrate, early nucleation of the second phase (α-Fe_2_O_3_) occurs, which substantially inhibits dislocations and ultimately leads to their complete disappearance as the thickness reaches 50 nm. These findings are consistent with those observed in YBaCuO films deposited on LaSrAlO_4_ substrates, where theoretical studies have shown that misfit stresses can induce phase transformations through the generation of misfit dislocations during the growth of cuprate films.^4^

**Fig. 4:**
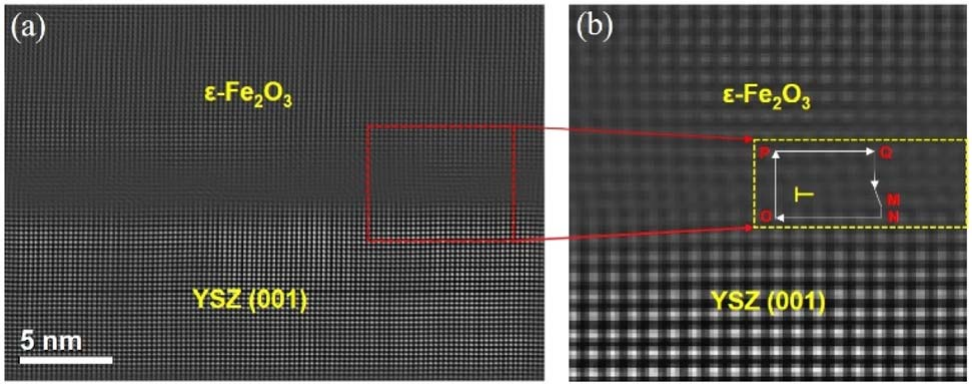
(a) Cross-sectional STEM-HAADF image of the ε-Fe_2_O_3_/YSZ(001) interface, showing the presence of dislocations in the ε-Fe_2_O_3_ phase. (b) Bragg-filtered STEM-HAADF image of the dislocation core (highlighted in the yellow box) extracted from the red box in (a). A Burgers circuit (O → P → Q → M → N → O) is constructed around the dislocation core. The closure failure of the circuit confirms the presence of an edge dislocation with a Burgers vector along the [001] direction.

The contrast variation observed in the BF-TEM image at the interface provides an important indication of the presence of misfit dislocations; however, this alone is insufficient to confirm their existence. To directly visualize misfit dislocations, STEM-HAADF imaging combined with Bragg filtering of selected atomic planes was performed on the ε-Fe_2_O_3_ film near a region exhibiting dark contrast (black dot), as shown in Fig. 4. The core of a misfit dislocation was clearly revealed within the yellow box in Fig. 4(b), extracted from the red-marked region in Fig. 4(a). Based on the analysis of a Burgers circuit around the dislocation core, the dislocation was identified as a [001]-type edge dislocation. The in-plane dislocation density δ* in the ε-Fe_2_O_3_ film (with a thickness of 20 nm) was estimated based on the number of such dislocation cores (black dots) along the interface, yielding a value of approximately 5 (dots)/22 (nm) = 22.7 × 10^5^ cm^−1^.

**Fig. 5:**
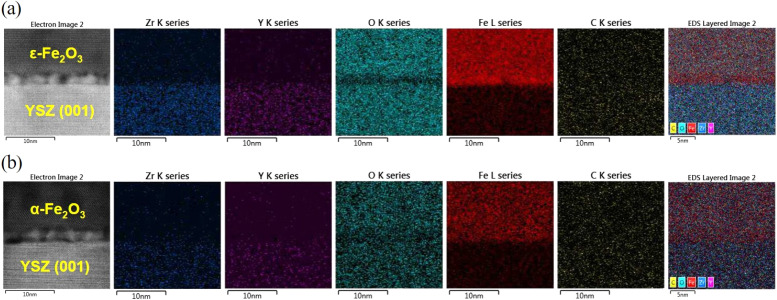
EDS elemental mapping for Zr, Y, O, and Fe in films with thicknesses of (a) 20 nm and (b) 50 nm. Bright colors indicate areas rich in each element, while darker regions denote areas with lower elemental presence.

To investigate the spatial distribution of elements in the film, energy-dispersive X-ray spectroscopy (EDS) measurements were performed on samples with thicknesses of 20 nm and 50 nm as shown in Fig. 5(a) and (b), respectively. Two distinct layers with a clear interface were observed, and the elements were represented by different colors. Notably, in the 20 nm film, the oxygen signal at the interface appeared darker than in the outer layer and substrate, suggesting the presence of oxygen vacancies. These results align with the dislocations observed in Fig. 3(a), (c) and 4, as misfit dislocations can lead to a higher concentration of oxygen defects at the interface compared to regions further away from it.^5^

Notably, high-resolution STEM-HAADF imaging and Bragg filtering were used to investigate the interface structure of the ε-Fe_2_O_3_/YSZ system. Although the interfacial region is only a few nanometers thick, the observed atomic stacking and lattice fringes appear consistent with the ε-Fe_2_O_3_ structure. Importantly, no lattice spacings corresponding to metallic Fe (*e.g.*, 0.203 nm for (110)) or FeO (*e.g.*, 0.301 nm for (200)) were observed. While EDS mapping suggests a degree of oxygen deficiency near the interface, this does not correspond to a distinct Fe or FeO phase but rather points to non-stoichiometry within the ε-phase. Due to the small number of atomic layers in this region, FFT analysis is inherently limited and should be interpreted with caution. However, the continuity of the ε-Fe_2_O_3_ lattice across the interface and the absence of secondary phases support the conclusion that oxygen loss leads to a partially reduced, non-stoichiometric ε-Fe_2_O_3_ phase. This behavior may be stabilized by misfit dislocations, which are known to accommodate strain and promote the formation of oxygen vacancies. Although our case differs from that reported by Matsuzaki *et al.*,^39^ where interfacial redox phenomena and oxygen diffusion effects in Fe_3_O_4_/YSZ significantly altered the oxide phase composition and stoichiometry, the comparison highlights the broader relevance of interfacial chemistry in oxide systems.

## Conclusions

In summary, the formation of misfit dislocations in Fe_2_O_3_ thin films at a critical thickness leads to the partial relaxation of misfit stresses, which induces phase transformations within the film. This creates a strong dependence of the film's phase composition on thickness, directly influencing the properties of iron(iii) oxide films, which are highly sensitive to their phase content. Controlling film thickness, therefore, allows precise manipulation of the phase composition, which is crucial for optimizing the functional properties of Fe_2_O_3_ thin films for specific applications. In the case of Fe_2_O_3_ films deposited on YSZ (001) substrates, our study shows that once the film reaches a critical thickness of around 20 nm, misfit dislocations form to relieve accumulated stress. As the thickness increases to 40–45 nm, these dislocations not only alleviate strain but also act as catalytic sites, promoting the formation of the α-Fe_2_O_3_ phase on the ε-Fe_2_O_3_ surface. Eventually, when the thickness reaches 50 nm, the ε-Fe_2_O_3_ phase fully transforms into α-Fe_2_O_3_. These findings highlight that controlling misfit dislocations and phase transformations through thickness adjustments offers a pathway to finely tune the material properties of Fe_2_O_3_ films. This ability is essential for enhancing the magnetic, electrical, and catalytic properties of iron oxide films, making them suitable candidates for applications in spintronics, sensors, and catalysis, where phase stability and functional performance are critical.

## Data availability

The authors confirm that the data supporting the findings of this study are available within the article. Raw data that support the findings of this study are available to the corresponding author, upon reasonable request.

## Author contributions

H.-J. Kim conceived the experiment's main idea. V.-H. Hoang prepared the samples, analysed the data, and wrote the original draft of the manuscript with assistance from H.-J. Kim. N.-S. Lee measured HR-TEM. All authors contributed to comments and revisions of the manuscript.

## Conflicts of interest

There are no conflicts to declare.
